# Supplementary figures and images for: Prevalence and virulence gene profiles of Escherichia coli O157 from cattle slaughtered in Buea, Cameroon
Source: PLoS One. 2020 Dec 15;15(12):e0235583. doi: 10.1371/journal.pone.0235583 (PMC7737970; doi:10.1371/journal.pone.0235583)

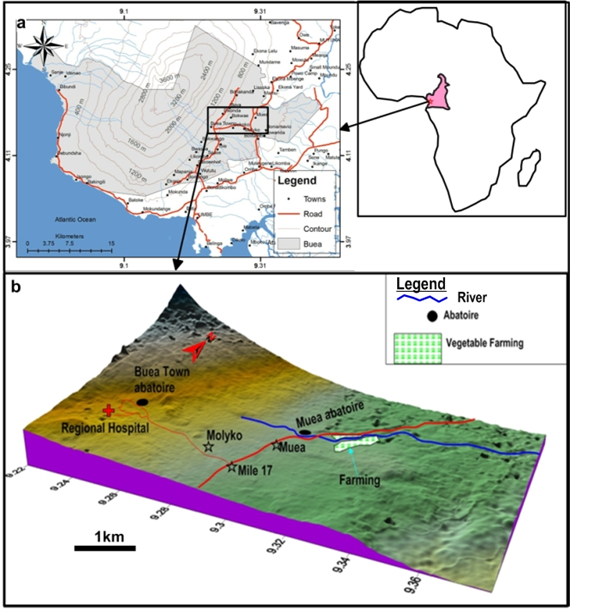

Supplement: S1 Fig — (TIF) [file pone.0235583.s001.tif]
