# Supplementary material for: Prevalence and virulence gene profiles of Escherichia coli O157 from cattle slaughtered in Buea, Cameroon
Source: PLoS One. 2020 Dec 15;15(12):e0235583. doi: 10.1371/journal.pone.0235583 (PMC7737970; doi:10.1371/journal.pone.0235583)
Supplement: Attachment — Submitted filename: PONE-D-20-18250_review.docx [file pone.0235583.s002.docx]

PONE-D-20-18250

Prevalence and Virulence Gene Profiles of *Escherichia coli* O157 from Cattle Slaughtered in Buea, Cameroon

This study analyzed 512 rectal samples from abattoirs for *E. coli* O157 using selective enrichment and isolation, followed by confirmation by latex agglutination and *rfb*-specific PCR. *E. coli* O157 was isolated from ~11% of the samples, and most of them carried *stx1* and/ *stx2*, along with *hlyA* and *eaeA* genes. The authors conclude that the cattle slaughtered for beef could be a potential threat to beef consumers in the region of their study.

This study reports what has already been reported from all over the world, and hence lacks novelty as such. The methods followed in this study do not add any advantage in terms of providing some new information or insight into the diversity, prevalence or virulence of *E. coli* O157 other than what is known about them. Hence, no new knowledge is generated. This study is vastly of local interest.

A brief analysis of the study reveals why no novel or interesting information is generated from this and similar studies. First, the isolation method used for O157 is conventional and does not have any added advantage in terms of improved isolation of typical or atypical strains of O157. Isolation of O157 based on sorbitol non-fermenting phenotype alone can underestimate their prevalence. Authors should also have tested equal number of sorbitol fermenting colonies. Concentration of O157 using immunomagnetic beads can improve the isolation of O157 including atypical strains. This is important since the study aims to understand the disease burden due to O157 through the consumption of beef.

The methodology followed for isolation is not clear. How many colonies were picked from each selective plate? The manuscript says “all NSF” colonies. This confusion extends to results section too (absence of line numbers in the manuscript makes it difficult to precisely point out the lines). In page 7, last paragraph, it is mentioned that 56 samples yielded *E. coli* O157, and all 56 colonies were confirmed by latex agglutination test and RfbO157 PCR. That’s 100 specificity of isolation, latex agglutination and PCR assays!. It is important to explain how many colonies were presumptively selected as *E. coli*, and how many of these were O157 and how many were not O157. When such investigations are done, multiple strains of same serotype/genotype are isolated, and these could be clonal or non-clonal. Comparison of these would be scientifically interesting and epidemiologically valuable.

The authors say that 10% prevalence is of great concern. However, it must be noted and as have authors mentioned in multiple places in the manuscript, that Shiga toxin-producing *E. coli* could be naturally associated with cattle, including healthy ones. In other words, STEC are not secondary contaminants in beef unlike vegetables, fish or chicken. In this context, it is also important to know the carriage/faecal shedding rate of O157 in healthy cattle, as well as in beef. If 10% is the prevalence rate in the abattoir, what percentage of beef in markets has these bacteria? Does faecal carriage simply indicate their prevalence in beef? These pertinent questions are not answered in this investigation.
