# Supplementary material for: Prevalence and virulence gene profiles of Escherichia coli O157 from cattle slaughtered in Buea, Cameroon
Source: PLoS One. 2020 Dec 15;15(12):e0235583. doi: 10.1371/journal.pone.0235583 (PMC7737970; doi:10.1371/journal.pone.0235583)
Supplement: Attachment — Submitted filename: Responses to Reviewers.docx [file pone.0235583.s003.docx]

**PLOS ONE**

**PONE-D-20-18250: Prevalence and Virulence Gene Profiles of *Escherichia coli* O157 from Cattle Slaughtered in Buea, Cameroon**

**Dear Editor, Reviewers,**

**Thank you for the review comments. Responses have been provided below along each review’s comments. We hope this is adequate.**

| Academic Editor | | |
| --- | --- | --- |
|  | Reviewer’s comment | Author’s comment |
| 1 | Please ensure that your manuscript meets PLOS ONE's style requirements, including those for file naming. The PLOS ONE style templates can be found at | Done: Manuscript format, naming of figures and tables has been adjusted |
| 2 | Please amend the manuscript submission data (via Edit Submission) to include author Seraphine Nkie Esemu, Achah Jerome Kfusi, Roland N. Ndip and Lucy M. Ndip. | Done |
| 3 | Your ethics statement must appear in the Methods section of your manuscript. If your ethics statement is written in any section besides the Methods, please move it to the Methods section and delete it from any other section. Please also ensure that your ethics statement is included in your manuscript, as the ethics section of your online submission will not be published alongside your manuscript. | Ethics statement has been moved to Methods section (page 5, lines 97 – 99) |
| Additional Editor Comments | Two reviewers have commented on the manuscript a and number of concerns have been raised. The authors need to revise the manuscript addressing all the comments point by point. The discussions should be in the light of recent references. The discrepancies in the number of colonies studied and confirmed needs to be corrected. Lack of testing of sorbitol fermenting colonies can be mentioned as a limitation of the study. | The manuscript has been revised as recommended by the reviewers (details below). Notably, the references have been updated to most recent, the discrepancy in the number of colonies confirmed has been addressed and non-inclusion of sorbitol fermenting colonies included as a limitation. |
| Reviewer 1 | | |
| Specific comments | Isolation of O157 based on sorbitol non-fermenting phenotype alone can underestimate their prevalence. Authors should also have tested equal number of sorbitol fermenting colonies. Concentration of O157 using immunomagnetic beads can improve the isolation of O157 including atypical strains. This is important since the study aims to understand the disease burden due to O157 through the consumption of beef. | The use of only Cefixime-Tellurite Sorbitol MacConkey (CT-SMAC) agar for isolation and the non-inclusion of sorbitol fermenting colonies has been stated as a limitation to the study (page 12 lines 248 – 250) |
|  | The methodology followed for isolation is not clear. How many colonies were picked from each selective plate? The manuscript says “all NSF” colonies. This confusion extends to results section too (absence of line numbers in the manuscript makes it difficult to precisely point out the lines). In page 7, last paragraph, it is mentioned that 56 samples yielded E. coli O157, and all 56 colonies were confirmed by latex agglutination test and RfbO157 PCR | Isolation of *E. coli* O157 in this study was on CT-SMAC while confirmation was with *E. coli* O157 latex agglutination test E. COLIPRO^TM^ O157 KIT and *E*. *coli* *rfb*_EO157_ PCR. The prevalence reported reflects only presumptive isolates that were confirmed. Unfortunately, the authors did not take interest in isolates that were not confirmed by PCR. This has been clarified (page 6, lines 117 – 119) and page 8, lines 161 – 163). |
|  | The authors say that 10% prevalence is of great concern. However, it must be noted and as have authors mentioned in multiple places in the manuscript, that Shiga toxin-producing E. coli could be naturally associated with cattle, including healthy ones. In other words, STEC are not secondary contaminants in beef unlike vegetables, fish or chicken. In this context, it is also important to know the carriage/faecal shedding rate of O157 in healthy cattle, as well as in beef. If 10% is the prevalence rate in the abattoir, what percentage of beef in markets has these bacteria? Does faecal carriage simply indicate their prevalence in beef? | This study which investigated the prevalence of *E. coli* O157 in cattle slaughtered in the Buea municipality is the first to report the pathogen in cattle in Cameroon as well as the first to confirm virulence genes associated with its pathogenicity in bovine isolates from this region. By also highlighting poor sanitary conditions in slaughter houses (lines 146 – 156 and Fig 1), we shed light on the public heath risk associated, especially as most human infections have been traced to contaminated beef. |
| **Reviewer 2** | | |
| Major comments | The review of literature is not recent. There are no references cited published after 2015 Only 14 references cited are published after 2010. | Most references have been updated |
|  | The authors should narrate some suggestions for improvement of abattoir conditions in Cameroon. | Recommendations for improvements of abattoir conditions in Cameroon have been included (page 12, lines 254 – 260). |
| Minor comments | Change Minutes to min and hours to h throughout the manuscript. Page 6, line 2: E. coil E. col | Theses have been corrected throughout the manuscript |
|  | Page 9, line 9: Our prevalence rate of 10.9% is of great concern for the entire country Change to : The prevalence rate reported in the present study……. | Done (Page 10, line 197) |
|  | Page 9, line 14: United State United States | Corrected (Page 10, line 200) |
|  | Fig 2. The names of the genes should be italicized. | Done (Fig 2) |
